# Beetle Species–Area Relationships and Extinction Rates in Protected Areas

**DOI:** 10.3390/insects11090646

**Published:** 2020-09-21

**Authors:** Simone Fattorini

**Affiliations:** Department of Life, Health and Environmental Sciences, University of L’Aquila, 67100 L’Aquila, Italy; simone.fattorini@univaq.it

**Keywords:** species–area relationship, elevational gradient, latitudinal gradient, reserves, biological conservation, extinction rates, Coleoptera, Mediterranean, Italy

## Abstract

**Simple Summary:**

Larger areas tend to host more species. This general ecological pattern (known as the species–area relationship, SAR) can be used to calculate expected extinction rates following area (habitat) loss. Here, using data from Italian reserves, SAR-based extinction rates are calculated for beetle groups with different ecology: terrestrial predators, aquatic predators, dung feeders, herbivores, and detritivores. Reserve area was an important predictor of species richness in all cases. However, also other factors besides area were important correlates of species richness. For some groups, species richness tends to decline with elevation and/or northwards. Extinction rates are higher for dung beetles, due to their dependence on large grazing areas, and detritivores, due to their low dispersal capabilities, which reduce their ability to reach new places when environmental conditions became less favorable. The lower extinction rates predicted for other groups can be explained by their higher dispersal ability. Extinction rates by area loss are always relatively low. This means that, in reserves with few species, many extinctions might be unnoticed.

**Abstract:**

The species–area relationship (SAR, i.e., the increase in species richness with area) is one of the most general ecological patterns. SARs can be used to calculate expected extinction rates following area (habitat) loss. Here, using data from Italian reserves, extinction rates were calculated for beetle groups with different feeding habits: Carabidae (terrestrial predators), Hydradephaga (aquatic predators), coprophagous Scarabaeoidea (dung feeders), phytophagous Scarabaeoidea (herbivores), and Tenebrionidae (detritivores). The importance of other factors besides area (namely latitude and elevation) was investigated. Reserve area was recovered as an important predictor of species richness in all cases. For Carabidae, Hydradephaga, and Tenebrionidae, elevation exerted a negative influence, whereas latitude had a negative influence on coprophagous Scarabaeoidea and Tenebrionidae, as a consequence of current and historical biogeographical factors. Extinction rates were higher for dung beetles, due to their dependence on large grazing areas, and Tenebrionidae, due to their low dispersal capabilities. The lower extinction rates predicted for Carabidae, phytophagous Scarabaeoidea, and Hydradephaga can be explained by their higher dispersal power. If other variables besides area are considered, extinction rates became more similar among groups. Extinction rates by area loss are always relatively low. Thus, in reserves with few species, many local extinctions might be unnoticed.

## 1. Introduction

The species–area relationship (SAR, i.e., the increase in species richness with area) is an almost ubiquitous ecological pattern [1]. There is increasing interest in how the SAR can be used for conservation purposes, including the selection of biodiversity hotspots [2,3,4], the identification of the best size and shape of natural areas [5,6,7,8], and the prediction of species extinction [9,10]. There is a debate about how accurate species extinction rates based on the SAR are [10]. Although some analyses suggest that SAR-based extinction rates are overestimated, empirical data indicate that the SAR probably underestimates, not overestimates species extinctions [9,10].

The basic idea in the use of the SAR to predict species loss is that a reduction in area size implies a reduction in species number. Thus, the same function which is used to model the SAR can be used, in a reverse way, to predict species loss. Empirical evidence largely supports that, at least for isolates, most systems are adequately modelled by the power function (1):

***S** = **cA**^**z**^* (1)

where ***S*** is the species number, ***A*** is area, and ***c*** and ***z*** are fitting parameters [11,12]. The power function is frequently applied in its linearized (log-log) form using decimal logarithms (2):

log(***S***) = log(***c***) + ***z*** log (***A***) (2)

In this form, ***c*** is the expected number of species per area unit, and ***z*** is the slope of the function [13].

The use of the SAR to predict species extinction is based on the assumption that if the original area ***A*_0_** is reduced to ***A*_1_**, the original number of species ***S*_0_** is expected to decline to ***S*_1_**, according to the following Equation (3) [10]:

***S*_1_** = ***S*_0_** (***A*_1_**/***A*_0_**)^***z***^ (3)

Using this approach, it is possible to predict extinction rates in isolated blocks of fragmented habitats, including protected areas, following area loss [14,15,16].

The Italian peninsula is located in the center of the Mediterranean Basin, one of the world’s hotspots of biodiversity [8,17,18], showing exceptionally high levels of diversity for a variety of plant and animal taxa [19,20]. At the same time, due to the profound, millenary impacts on the wilderness of this area, the Mediterranean biodiversity has been strongly influenced by the human presence, and it is currently under many threats [21,22].

According to the last official report, in Italy there are some 900 protected areas, occupying a terrestrial surface of about three million hectares (about 11% of the country) [23], plus about other 400 areas that benefit from some form of protection, for a further 430,000 hectares [24]. The number and size of protected areas is, however, under continuous change.

Biodiversity knowledge for most of the Italian protected areas is extremely poor, especially for invertebrates. However, for at least some groups, there is information about the number of species present in some reserves. In particular, thanks to the interest of professional and amateur entomologists, some beetle groups are among the few invertebrates for which it is possible to gather reliable values of species richness for sets of Italian reserves. Beetles are the most diversified group of living organisms, constituting about 40% of all described insect species and 25% of all known life-forms [25,26]. Beetles occur in most terrestrial and freshwater habitats and are ecologically extremely diversified, which makes these insects very useful in comparative analyses: for example, with reference to their trophic habits, dispersal capabilities, and ecological preferences.

In this paper, I used data on beetle groups characterized by very different ecology (terrestrial predators, aquatic predators, dung feeders, herbivores, and terrestrial detritivores) from Italian reserves to investigate how extinction rates based on the use of the SAR vary according to the beetle’s ecology.

## 2. Materials and Methods

I collected literature data on species richness for Carabidae, Hydradephaga, Scarabaeoidea, and Tenebrionidae from Italian reserves. Carabidae are a family of mainly terrestrial predators [27], whereas Hydradephaga are a group of families (Gyrinidae, Haliplidae, Noteridae, Dytiscidae, Hygrobiidae for the Italian fauna) of freshwater predators [28,29]. Scarabaeoidea, as intended here, include Trogidae, Bolboceratidae, Geotrupidae, Hybosoridae, Ochodaeidae, Glaphyridae, and Scarabaeidae (including Aphodiinae, Scarabaeinae, Orphninae, Melolonthinae, Rutelinae, Dynastinae, and Cetoniinae), but not Lucanidae [30]. The Scarabaeoidea include species with a wide spectrum of feeding habits, but most of the species are either coprophagous (dung beetles) or phytophagous [30]. For this reason, I conducted analyses for the whole group and for the coprophagous and phytophagous species separately (the whole group analyses included more species than the sum of coprophagous and phytophagous because of the presence of species that did not belong to these two categories, for example mycetophagous species). Finally, Tenebrionidae are essentially saprophagous insects [27]. Although the Alleculinae (formerly considered a separate family) fall within the tenebrionid cladogenesis, I did not consider them in the analysis because of their extremely different and highly derived ecological characteristics (they are the only flower visiting tenebrionids and, contrary to most tenebrionids, they are flying insects) [31]. Additionally, for most reserves, there is no information on alleculines. I also omitted the genus *Lagria* (another flower visiting tenebrionid) and the genus *Myrmechixenus*, since its distribution is very poorly known. I also omitted synanthropic species [31].

**Figure 1:**
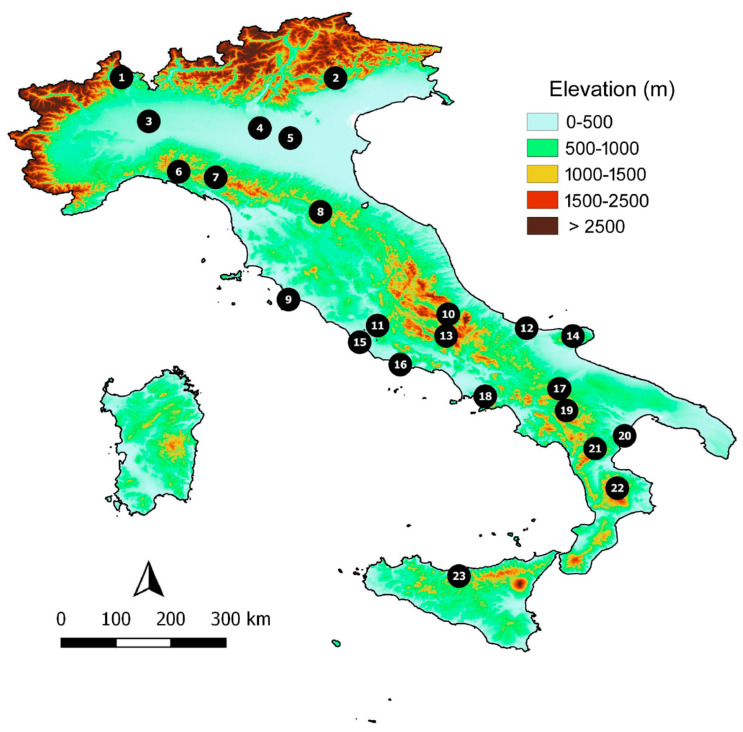
Location of the 23 Italian reserves considered in this study. Reserve names are given in Table 1.

Overall, I collected data from 23 Italian reserves (18 reserves for Carabidae [32,33,34,35,36,37,38,39,40,41,42,43,44,45], 14 reserves for Hydradephaga [32,33,35,46,47,48,49,50], 18 reserves for Scarabaeoidea [32,33,35,37,49,51,52,53,54,55,56,57,58,59], and 18 reserves for Tenebrionidae [32,33,35,37,54,60,61,62,63,64,65,66,67,68,69,70,71] (Figure 1). Contiguous reserves were considered to a be a single reserve. Values of species richness for the various beetle groups in each reserve are given in Table 1.

For reserve areas, where possible, I referred to the official size. Some study areas did not correspond exactly to the borders of a reserve. This occurred when a study area was only a part of a larger reserve (reserve 22), when the study area was the sum of contiguous reserves (reserves 14 and 15), or when the study area included adjacent areas (reserves 10, 13, 23). In such cases, I used as the size of the study area the surface reported in the reference used to extract the faunal data; if not given, I calculated this surface from the maps provided by the authors of the faunal studies.

The selected reserves ranged from 0.17 to 1700 km^2^, thus spanning for four orders of magnitude. This avoided the risk of the SAR appearing to be linear because of sampling a small range of areas [72]. Reserves were distributed throughout the Italian peninsula and Sicily to fully encompass the Italian latitudinal gradient. Reserves were also representative of coastal, internal plain, and montane landscapes.

SARs were first modelled using the linearized version of the power function (Equation (2)) with log_10_. Values of ***c*** and ***z*** for the SAR of the various groups were compared using Analysis of Covariance (ANCOVA) [13]. ***z***-values of SARs were used to calculate the expected number of species lost with increasing area loss using Equation (3).

Although this is the standard procedure to infer potential species loss due to area reduction, the number of species is not controlled only by area. Even when area is the most important determinant of species richness, other variables may exert a relevant role. Elevation is an important macroecological variable that influences species richness [73]. To take into account the role of elevation, for each area, I considered various measures of elevation: minimum, maximum, average, and range. Elevational range was considered as a proxy of environmental variability. Mean elevation was intended to reflect the overall orographic physiognomy of a given area, whereas minimum and maximum elevations were introduced because extreme values are clearly distinctive of coastal and montane areas.

When elevation of sampling sites was reported in the examined faunal studies, their values were used to calculate minimum elevation, maximum elevation, elevational range (maximum minus minimum) and mean elevation for the study areas (reserve 1 [43], reserves 6 and 7 [74], reserve 14 [75], reserve 18 [76], reserve 21 [77], reserve 22 [78]). For reserve 8, I used elevation of sampling points reported by Lestes [36] for Carabidae and by Rocchi and Mascagni [50] for Hydradephaga. For reserve 5, I referred to the minimum, maximum, and mean values reported by the authors of the faunal study [54]. For reserve 23, I calculated the average elevation using minimum and maximum values as given by Aliquò and Leo [60], integrated with topographic maps. In other cases, I calculated these values from topographic maps.

Due to the peculiar, north–south orientation of Italy, study areas are distributed along a latitudinal gradient. It is well known that species richness of most taxa shows a distinct latitudinal pattern, decreasing (in the northern hemisphere) from south to north [79,80,81,82,83]. This might influence the SAR by negatively affecting species richness in northern areas. For this reason, I also considered the latitude (measured in decimal degrees) of the reserve centroid as a further correlate. All variables were log_10_-transformed prior to analyses; a log_10_(*x*+1) transformation was used to manage zero values for coprophagous Scarabaeoidea (because of the presence of a reserve with no species) and minimum elevation (for coastal areas with a minimum elevation of 0 m).

To evaluate the relative importance of elevation (minimum, maximum, mean, and range) and latitude in determining species richness besides reserve area, I used a multimodel selection procedure in which all variables were tested individually and in all their possible combinations. Then, models were ordered by decreasing values of the small-sample corrected Akaike Information Criterion (AICc), and the model with the lowest AICc was selected as the best model; alternative models with ΔAIC values ≤ 2 were considered as equally supported [84]. When the best fit model included other variables than area, the coefficient of area in the multiple model was used as a *z*-value for the estimation of species loss in equation 3. In practice, this approach consists in predicting species loss by area reduction, holding stable the influence of all other variables with an exponent *z* that, however, takes into account their influence in species richness. This is reasonable in ecological terms, since the surface of a certain study area suitable for a certain group of species can be reduced by habitat fragmentation, loss, or alteration, whereas its geographical position and orography remain unchanged.

All analyses were conducted in R version 3.5 [85]. Multimodel selection was performed using the library MuMIn [86].

## 3. Results

**Figure 2:**
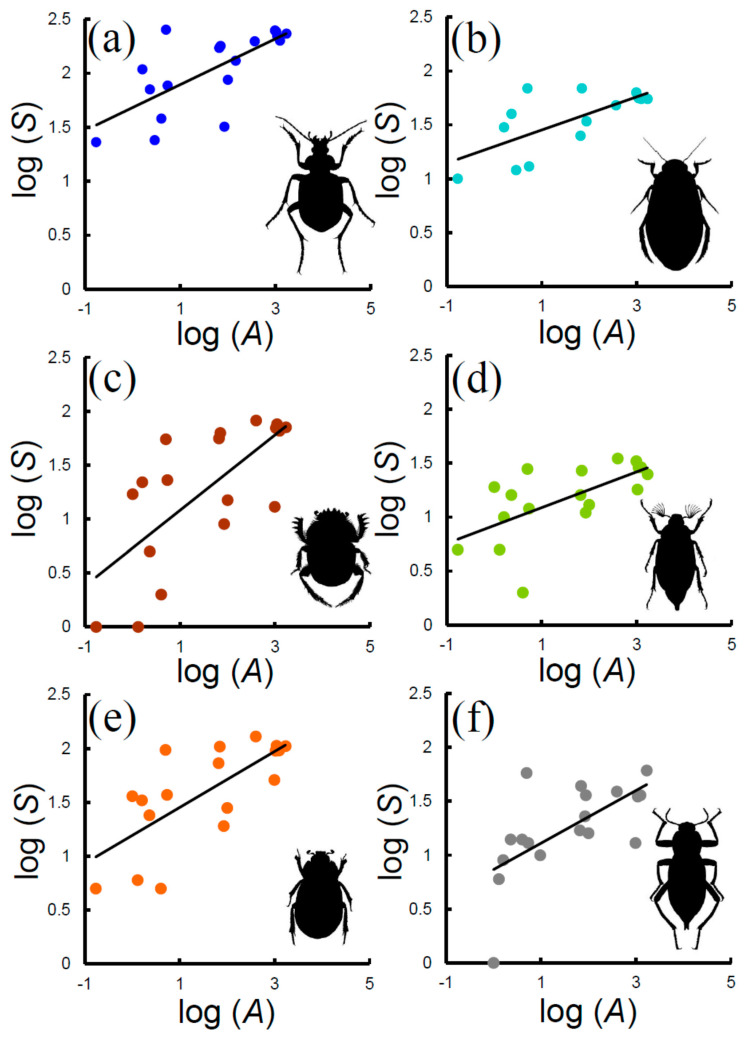
Species–area relationships (SARs) for various beetle groups in Italian reserves: (**a**) Carabidae, (**b**) Hydradephaga, (**c**) coprophagous Scarabaeoidea, (**d**) phytophagous Scarabaeoidea, (**e**) total Scarabaeoidea, (**f**) Tenebrionidae. Decimal logarithms are used.

The power function modelled SARs of the various groups with very similar goodness of fit (Figure 2, Table 2). *z*-values, which indicate the rate at which species accumulate with area, did not vary significantly between groups (ANCOVA: *F* = 1.536, *P* = 0.200). By contrast, ANCOVA indicated an overall significance difference between *c*-values (*F* = 18.72, *P* << 0.001). *c*-values, which represent the expected number of species per area unit, decreased in the following order (~indicates that differences in *c*-values were not significant in pairwise ANCOVAs): Carabidae (48 species) < Hydradephaga (20) < phytophagous Scarabaeoidea (8) ~ Tenebrionidae (7) ~ coprophagous Scarabaeoidea (5).

The results of multimodel selection (Table 3) showed that area was an important predictor in all cases and, for both total and phytophagous Scarabaeoidea, it was the only variable included in the best fit models.

For Carabidae, two equally supported models (ΔAICc = 1.13) were selected as the best fit models: the first model included area (positively) and elevational range (negatively, but not significantly); the second model included only area (positively). For Hydradephaga, the best fit model included area (positively) and elevational range (negatively). For coprophagous Scarabaeoidea, the best fit model included area (positively) and latitude (negatively). For coprophagous and all Scarabaeoidea, the best fit models included only area (positively). Finally, for Tenebrionidae, the best fit model included area (positively), latitude (negatively), and minimum elevation (negatively).

**Figure 3:**
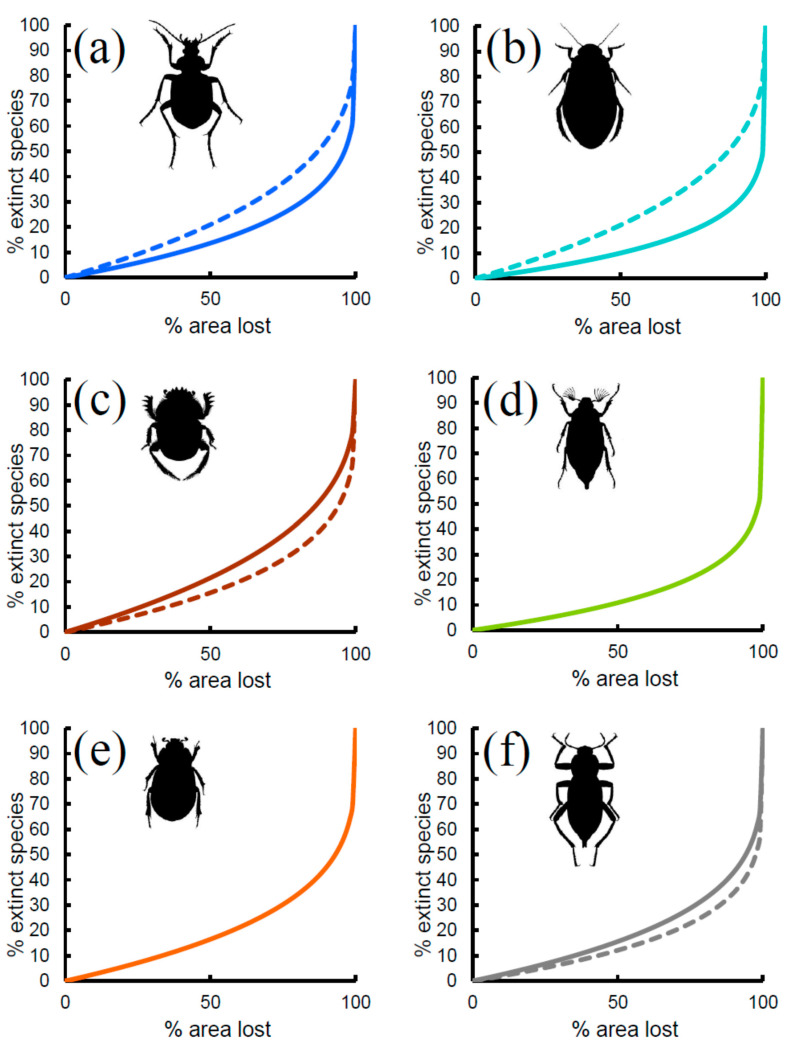
Extinction rates (% of extinct species) expected on the basis of area loss (% of area lost) for various beetle groups in Italian reserves: (**a**) Carabidae, (**b**) Hydradephaga, (**c**) coprophagous Scarabaeoidea, (**d**) phytophagous Scarabaeoidea, (**e**) total Scarabaeoidea, (**f**) Tenebrionidae. Solid lines: expected rates from the species–area relationship. Dashed lines: expected rates from multiple regression models.

Predicted rates of species extinction based on the ***z***-values of the power function (Figure 3, solid lines) showed similar patterns among groups, with small proportions of extinct species until the fraction of lost area was very large. The most sensitive group was the coprophagous Scarabaeoidea, for which a loss of 25% of area would lead to the local extinction of about 9% of the species (13% of species with 33% of area lost, and 21% with 50% of area lost, respectively). Tenebrionidae would lose about 7% of species if 25% of area was lost (9% of species with 33% of area lost, and 15% with 50% of area lost, respectively). Total Scarabaeoidea showed a very similar trend. Carabidae would lose about 6% of species if 25% of area was lost (8% of species with 33% of area lost, and 13% with 50% of area lost, respectively). Phytophagous Scarabaeoidea would lose about 5% of species if 25% of area was lost (6% of species with 33% of area lost, and 11% with 50% of area lost, respectively), and a virtually identical pattern was found for the Hydradephaga. Thus, sensitivity to extinction by area reduction increased in the order: coprophagous Scarabaeoidea < Tenebrionidae < Carabidae < phytophagous Scarabaeoidea < Hydradephaga.

**Figure 4:**
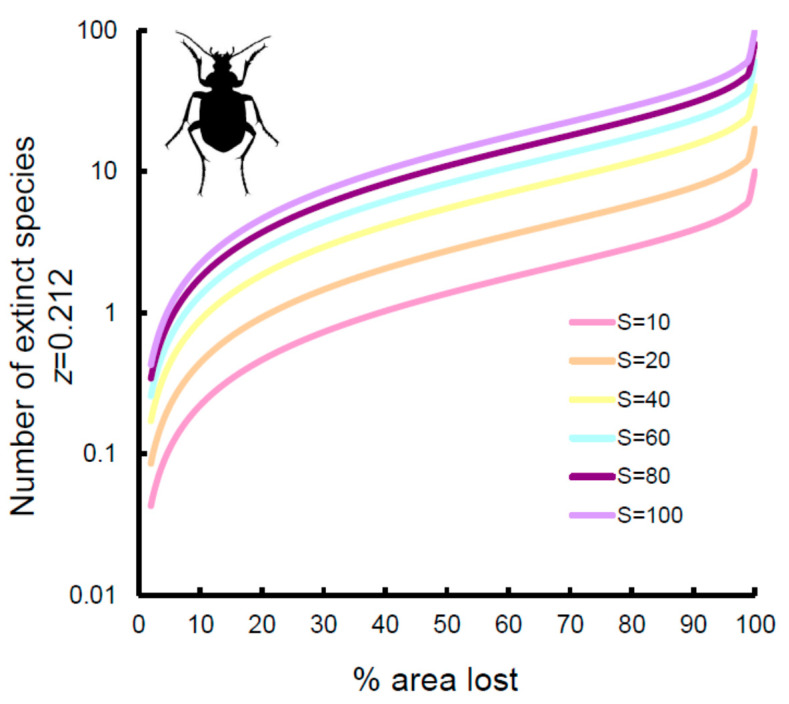
Number of extinct species expected on the basis of area loss (% of area lost) for the Carabidae of Italian reserves for areas hypothetically hosting 10, 20, 40, 60, 80, and 100 species. Curves were calculated using ***z*** = 0.212, as obtained from the power function of the species–area relationship. Use of ***z***-values from multiple models did not change the pattern. Note the log-scale for the ***y***-axis.

If the coefficient for area obtained from the multiple regression models was used (Figure 3, dashed lines), extinction rates increased substantially for Carabidae and Hydradephaga, with virtually identical trends (9% of species with 25% of area lost, 12% of species with 33% of area lost, and 21% with 50% of area lost, respectively). By contrast, extinction rates decreased slightly for coprophagous Scarabaeoidea (7% of species with 25% of area lost, 9% of species with 33% of area lost, and 15% with 50% of area lost, respectively) and Tenebrionidae (5% of species with 25% of area lost, 7% of species with 33% of area lost, and 12% with 50% of area lost, respectively). However, because of the relatively small number of species occurring in each reserve, extinction might be difficult to detect. As shown in Figure 4 for Carabidae (other groups had virtually identical patterns; the use of *z*-values from multiple model also did not change the patterns; see Figure S1), with ten species, an area loss of more than 25% is necessary to observe one extinction, whereas with 100 species, a loss of 3% is sufficient to have one extinction. A reduction of 33% of area would still translate to the loss of one species in a reserve containing 10 species, 5 species in a reserve with 40 species, and 13 species in a reserve with 100 species. This suggests that extinctions might be hardly detectable, even for a very large loss of area, if the reserve is small and contains few species.

## 4. Discussion

The *z*-values of the power function SARs found in this study (*z* = 0.15–0.35) were perfectly in the range typically found for isolates (*z* = 0.2–0.4; [11,12]). The *c*-values indicate that Carabidae had much more species per area unit than any other group. The second group with the highest number of species per area unit is the Hydradephaga. Phytophagous and coprophagous Scarabaeoidea and Tenebrionidae have similar *c*-values, much lower than those for Carabidae and Hydradephaga. It is an unexpected result that predators have more species than dung feeders, herbivores, and detritivores. However, it is known that *c*-values reflect species dispersal abilities, being larger for groups with higher dispersal abilities [13]. Thus, it is possible that the high number of species per area unit in Carabidae and Hydradephaga reflect their higher dispersal abilities. Carabids are known to have, in general, high dispersal capabilities, especially the macropterous forms [87,88,89]. Similarly, many Hydradephaga are known to fly for long distances and move between waterbodies, which is essential for animals that must survive in lentic waterbodies subject to seasonal drought [90]. By contrast, the small number of tenebrionid species per area unit can be related to the typically low dispersal power of these mainly flightless beetles [91,92,93]. The small value of species richness per area unit of phytophagous Scarabaeoidea can be interpreted as a result that some species are associated with some plants or forms of vegetation, and hence high values of species richness can be found only in complex landscapes. Coprophagous Scarabaeoidea depend on the presence of dung, which is in turn typically associated with the presence of cattle; this means that large grazing areas are needed to host a rich fauna. The importance of area for both coprophagous and phytophagous Scarabaeoidea is further shown by the fact that this variable overwhelms the contribution of all other tested variables, being the only one retained in the multimodel selection procedure.

For Hydradephaga and possibly Carabidae, in addition to area, an important contribution in explaining species richness is given by the elevational range, which, however, exerts a negative influence. Elevational range is usually considered as a measure of environmental heterogeneity, because it is correlated with variation in temperature, precipitation, humidity, wind speed, evaporation, and insolation, and altitude is often found to be an important variable in explaining species numbers on islands, in some cases ranking only second to, or even ahead of, island area [73]. On the contrary, in the present research, elevational range had a negative influence, albeit only in the case of Hydradephaga and possibly Carabidae. This may be interpreted as a consequence of the fact that, in a mainland context, a large elevational gradient implies that most of the study area is at high altitudes. Since, in general, richness tends to decrease with elevation [94,95,96], the negative influence of elevational range may be a reflection of this general trend. Moreover, in the specific case of the Hydradephaga, the negative impact of the elevational range on species richness may be explained by the fact that, with increasing elevation, lentic waters became rare. Elevation exerted a negative influence also on Tenebrionidae, but, in this case, the variable retained in the multiple model was minimum elevation. The negative impact of minimum elevation on species richness can be explained by the fact that many tenebrionids are associated with coastal areas [97].

In accordance with the general impoverishment of species richness with increasing latitude observed for many taxa in the northern hemisphere [79,80,81,82,83], latitude had a negative influence on Tenebrionidae and coprophagous Scarabaeoidea. This can be explained with reference to both current climatic factors and biogeographical history. Tenebrionidae are mainly thermophilic animals, including many groups associated with hot and arid environments [27,97], and their species richness in Europe tends to decrease northwards due to the joint effect of current climatic factors and post glaciation incomplete recolonization [98,99,100]. In accordance with the general latitudinal pattern of biodiversity in Europe, tenebrionid richness in Italy is known to increase southwards [16], in response to variations in rainfall and temperatures, and in consequence of the refugial role played by Southern Italy during the Pleistocene [101]. There is no previous study on the latitudinal gradient of dung beetles in Europe or in Italy, but studies on dung beetle communities in Europe and North America [102,103,104] indicate that northern communities have fewer species than the southern ones. The increase in dung beetle richness with decreasing latitude in Italy can be explained by both historical reasons (i.e., the refugial role of southern areas during Pleistocene glaciations) and current climate (for example, while Aphodiini are able to colonize even cold areas, most Scarabaeinae are thermophilic animals) and land use (extensive grazing in southern Italian regions).

Extinction rates as predicted by the SAR are highest for dung beetles, which is consistent with their dependence on dung, which, in turn, is mainly associated with the presence of large grazing areas. Actually, dung beetles were the insect group which experienced the highest documented extinction rate in the city of Rome as a consequence of the loss of grazing lands due to urbanization [105]. The second most sensitive group was the Tenebrionidae, probably due to their low dispersal capabilities, which reduce their possibility of colonizing new areas when environmental conditions become less favorable. A previous study focused on these insects revealed that the geophilous tenebrionids, which are typically flightless, have higher extinction rates than the xylophilous ones [16]. Moreover, Tenebrionidae include many saproxylic beetles, which are particularly sensitive to the loss of forest vegetation [106,107]. The comparatively low extinction rates predicted for Carabidae, phytophagous Scarabaeoidea and Hydradephaga can be explained by their higher dispersal power, which reduces habitat confinement and hence makes them less sensitive to area loss, despite their dependence on other animals as food (Carabidae), plants (phytophagous Scarabaeoidea), or specific biotopes (lentic water for Hydradephaga). If other variables besides area are considered in multiple models, however, the extinction rates expected by area loss became more similar among groups, suggesting that differences in extinction rates are largely due to the influence of other factors than area. Including the effect of latitude in multiple models reduces the expected extinction rates for Tenebrionidae and coprophagous Scarabaeoidea, which highlights a prominent role of the southern areas for their conservation in Italy. In other words, for these beetles, area loss is expected to act more dramatically in southern areas. By contrast, for Carabidae and Hydradephaga, the inclusion of elevational range increased the extinction risk, which suggests that lowland areas are those that most contribute to the conservation of these beetles. Finally, the extinction rates expected by area loss are always relatively low. Thus, in small areas hosting relatively few species, a very small number of species will be lost, even if they represent a large proportion of the considered fauna. This means that, because of difficulties in obtaining complete species lists and in monitoring all species, in small reserves, with few species, the number of possible extinctions might be too small to be detected, and extinctions might remain unnoticed.

## 5. Conclusions

Reserve area was an important predictor of species richness in all investigated beetle groups, which is consistent with the almost ubiquitous pattern known as the species–area relationship (SAR). For Carabidae, Hydradephaga, and Tenebrionidae, elevation exerted a negative influence—a possible reflection of the general decline in species richness with altitude commonly observed in many taxa. In accordance with the general impoverishment of species richness northwards, latitude had a negative influence on coprophagous Scarabaeoidea and Tenebrionidae, as a result of current and past biogeographical factors (that is, more favorable climatic conditions in southern areas and their role as Pleistocene refuges). SAR-based extinction rates are highest for dung beetles, consistently with their dependence on large grazing areas, and for Tenebrionidae, because of their low dispersal capabilities. The relatively lower extinction rates predicted for Carabidae, phytophagous Scarabaeoidea, and Hydradephaga can be explained by their higher dispersal power. Thus, both ecological needs and dispersal abilities emerge as important determinants of SAR-based extinction rates. If other variables besides area are considered, extinction rates become more similar among groups. Because of the overall low extinction rates, extinctions might be too few to be easily detected in reserves with few species. This should be carefully considered when comparing SAR-based extinction rates with empirical data.

## Figures and Tables

**Table 1:** Number of species for various beetle groups in Italian reserves. Res: Reserve number ^1^ (reserves are numbered as in Figure 1); Lat: Latitude (decimal degrees); Lon: Longitude (decimal degrees); Area: reserve size (km^2^); Min: Minimum elevation (m); Max: Maximum elevation (m); Range: Elevational range (m); Mean: Mean elevation (m). Beetle groups: Ca: Carabidae; Hy: Hydradephaga; Co: coprophagous Scarabaeoidea; Ph: phytophagous Scarabaeoidea; Sc: all Scarabaeoidea; Te: Tenebrionidae. For reserve 8, elevational data without parentheses refer to Carabidae sampling points, and those in parentheses to Hydradephaga sampling points.

Res	Lat	Lon	Area	Min	Max	Range	Mean	Ca	Hy	Co	Ph	Sc	Te
1	46.03	8.48	146	400	2200	1820	1116.5	131	-	-	-	-	-
2	46.02	11.98	1	225	233	8	229	-	-	16	19	36	1
3	45.31	8.93	983	116	300	184	208	249	63	12	33	51	13
4	45.20	10.74	2.3	22	26	4	24	71	40	4	16	24	14
5	45.04	11.24	1.3	7	15	8	14	-	-	0	5	6	6
6	44.49	9.42	0.17	1326	1350	24	1330.5	23	10	0	5	5	-
7	44.40	10.02	2.9	1330	1470	140	1406.5	24	12	-	-	-	-
8	43.83	11.73	368.5	550 (350)	1200 (1450)	650 (1106)	868 (753.5)	198	48	-	-	-	-
9	42.40	11.21	4	0	3	3	1.5	38	1	1	2	5	14
10	42.16	13.82	100	300	400	100	350	87	-	14	13	28	16
11	41.98	12.67	5.4	50	120	70	85	77	13	22	12	37	13
12	41.93	15.11	9.6	0	1	1	0.5	-	-	-	-	-	10
13	41.81	13.79	1040	700	2249	1549	1474.5	243	-	69	18	95	-
14	41.81	15.85	1700	0	950	950	456	233	55	70	25	105	61
15	41.70	12.38	70	0	70	70	35	179	69	62	27	104	44
16	41.34	13.04	88	0	541	541	270.5	-	34	-	-	-	36
17	40.95	15.63	66	650	1326	676	988	172	25	55	16	73	17
18	40.82	14.43	84.8	200	1281	1081	740.5	32	-	8	11	19	23
19	40.59	15.75	1.6	764	770	6	767	109	30	21	10	33	9
20	40.18	16.70	5	0	6	6	3	253	69	54	28	97	58
21	39.97	16.21	1100	800	2000	1200	1482	220	56	75	29	106	35
22	39.32	16.58	1250	1000	1900	900	1425	201	55	65	29	96	36
23	37.89	14.00	399.4	400	1979	1579	1189.5	-	-	81	35	129	39

^1^ Reserve names: 1: Val Grande National Park; 2: Vincheto di Celarda Nature Reserve; 3: Ticino Regional Park; 4: Bosco Fontana Nature Reserve; 5: Isola Boscone Nature Reserve; 6: Agoraie Nature Reserve; 7: Guadine Pradaccio Nature Reserve; 8: Foreste Casentinesi National Park; 9: Burano Nature Reserve; 10: Pescara Spring Nature Reserve and adjacent areas in the Valle Peligna; 11: Inviolata Archeological and Nature Park; 12: Foce Saccione Site of Community Importance; 13: Abruzzo, Latium and Molise National Park; 14: Gargano National Park and Isola Varano Nature Reserve; 15: Castelporziano Presidential Estate and Castelfusano Urban Park; 16: Circeo National Park; 17: Vulture Natural Park; 18: Vesuvius National Park; 19: Pantano di Pignola Reserve; 20: Policoro Reserve; 21: Pollino National Park; 22: Sila National Park; 23: Madonie Regional Natural Park.

**Table 2:** Species–area relationships (SARs) for beetles in Italian reserves. SARs were modelled using the linearized version of the power function with decimal logarithms: log(*****S*****) = log(***c***) + ***z*** log (***A***), where ***S*** is the species number, ***A*** is area, and ***c*** and ***z*** are fitting parameters. SE: standard error, ***t*** = Student’s ***t***, ***p*** = probability, ***R*^2^** = goodness of fit.

Beetle Group, ***R*^2^** Values, and Estimated Parameters	Estimate ± SE	*t*	*p*
Carabidae (***R***^2^ = 0.50)			
log(***c***)	1.68 ± 0.11	15.65	<<0.001
***z***	0.21 ± 0.05	4.03	<0.001
Hydradephaga (***R***^2^ = 0.47)			
log(***c***)	1.30 ± 0.10	13.65	<<0.001
***z***	0.15 ± 0.05	3.26	0.007
Scarabaeoidea (coprophagous) (***R***^2^ = 0.49)			
log(***c***)	0.73 ± 0.18	4.15	<<0.001
***z***	0.35 ± 0.09	3.93	0.001
Scarabaeoidea (phytophagous) (***R***^2^ = 0.44)			
log(***c***)	0.92 ± 0.10	9.41	<<0.001
***z***	0.17 ± 0.05	3.37	0.004
			
Scarabaeoidea (total) (***R***^2^ = 0.51)			
log(***c***)	1.19 ± 0.13	9.44	<<0.001
***z***	0.26 ± 0.06	4.08	<0.001
			
Tenebrionidae (***R***^2^ = 0.42)			
log(***c***)	0.87 ± 0.14	6.26	<<0.001
***z***	0.25 ± 0.07	3.38	0.004

**Table 3:** Best fit models for the influence of area, latitude, and elevation on beetle richness in Italian reserves. Min: Minimum elevation (m); Range: Elevational range (m); df: degrees of freedom; ***R***^2^_adj_: adjusted ***R***^2^; AICc: corrected Akaike Information Criterion. Errors refer to standard errors. * = ***p*** <0.05, ** = ***p*** < 0.01, *** ***p*** < 0.001.

	Intercept	Area	Latitude	Min	Range	df	*R* ^2^_adj_	AICc
Carabidae								
	1.90 ± 0.15 (***)	0.34 ± 0.08 (***)			−0.20 ± 0.10	4	0.56	7.7
	1.68 ± 0.11 (***)	0.21 ± 0.05 (***)				3	0.47	8.9
Hydradephaga								
	1.71 ± 0.09 (***)	0.34 ± 0.04 (***)			−0.34 ± 0.06 (***)	4	0.84	−11.2
Scarabaeoidea (coprophagous)								
	19.74 ± 8.21 (*)	0.25 ± 0.09 (*)	−11.61 ± 5.01 (*)			4	0.58	27.8
Scarabaeoidea (phytophagous)								
	0.92 ± 0.10 (***)	0.17 ± 0.05 (**)				3	0.38	8.7
Scarabaeoidea (total)	1.19 ± 0.13 (***)	0.26 ± 0.06 (***)				3	0.48	18.0
Tenebrionidae								
	17.97 ± 4.14 (**)	0.19 ± 0.05 (**)	−10.34 ± 2.52 (**)	−0.16 ± 0.04 (**)		5	0.76	5.2

## Supplementary Materials

The following are available online at https://www.mdpi.com/2075-4450/11/9/646/s1, Figure S1: number of extinct species expected on the basis of area loss (% of area lost) for various groups of beetles in Italian reserves for areas hosting 10, 20, 40, 60, 80, and 100 species. Curves were calculated using *z*-values obtained from the power function of the species–area relationship and from multiple models. Note the log-scale for the y-axis. (**a**) and (**b**) Carabidae; (**c**) and (**d**) Hydradephaga; (**e**) and (**f**) coprophagous Scarabaeoidea; (**g**) phytophagous Scarabaeoidea; (**h**) total Scarabaeoidea; (**i**) and (**j**) Tenebrionidae. Panels (**a**), (**c**), (**e**), (**g**), (**h**), and (**i**) refer to *z*-values obtained from the power function. Panels (**b**), (**d**), (**f**), and (**j**) refer to *z*-values obtained from multiple models.
