# Supplementary figures and images for: Beetle Species–Area Relationships and Extinction Rates in Protected Areas
Source: Insects. 2020 Sep 21;11(9):646. doi: 10.3390/insects11090646 (PMC7563763; doi:10.3390/insects11090646)

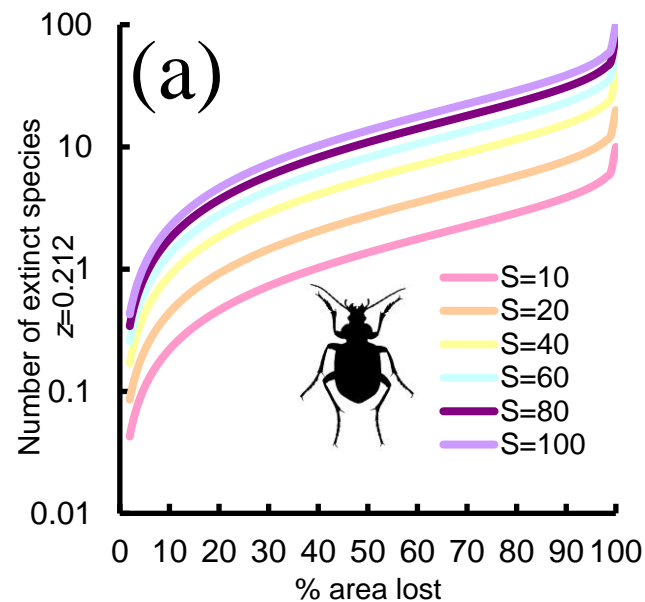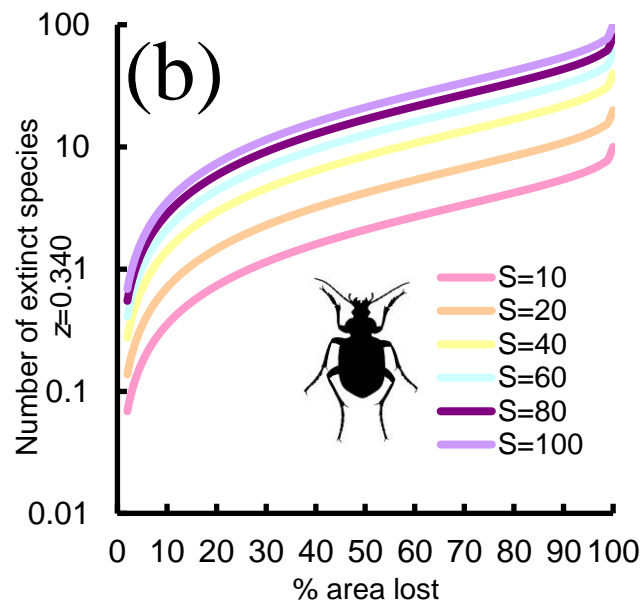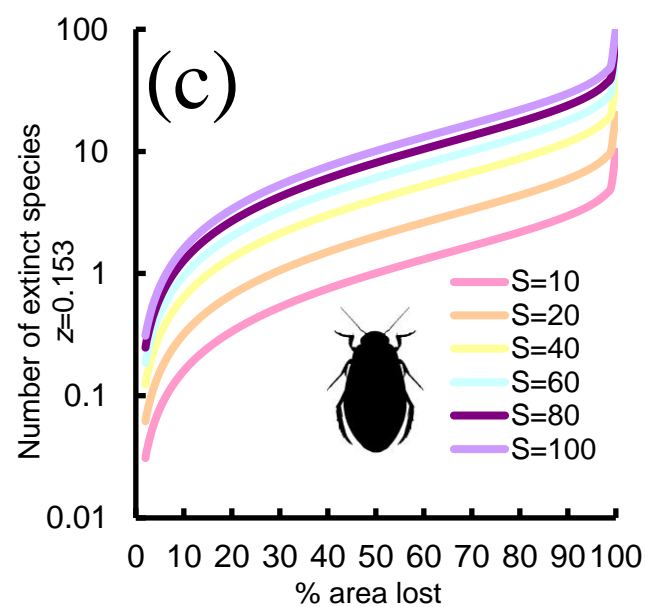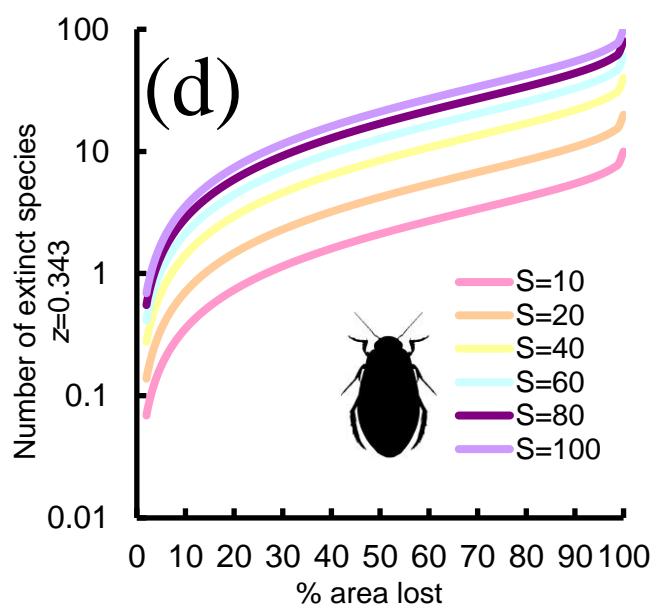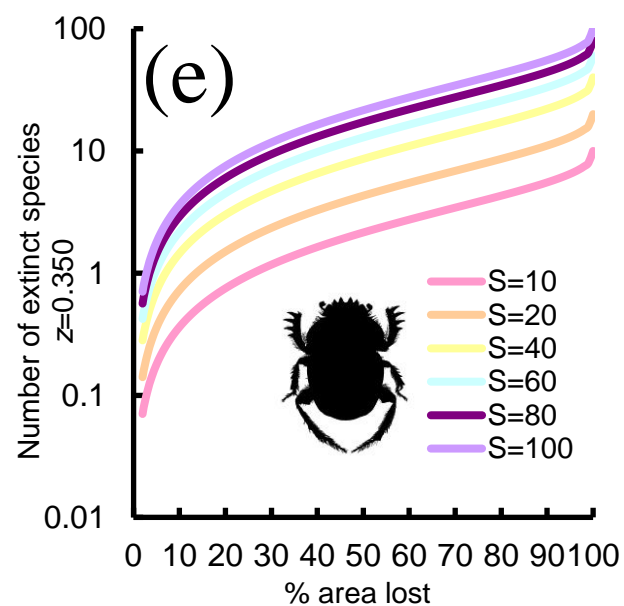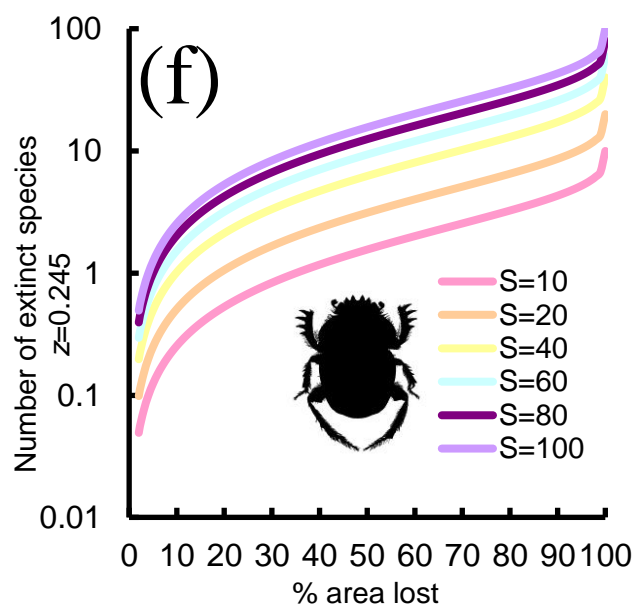

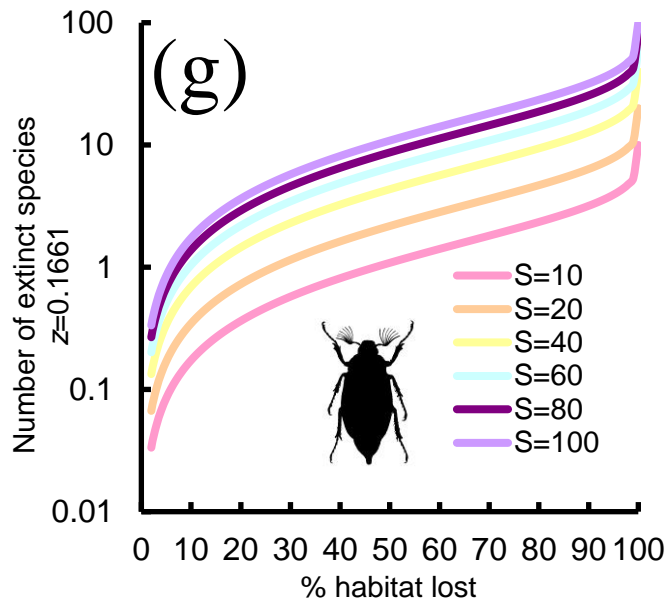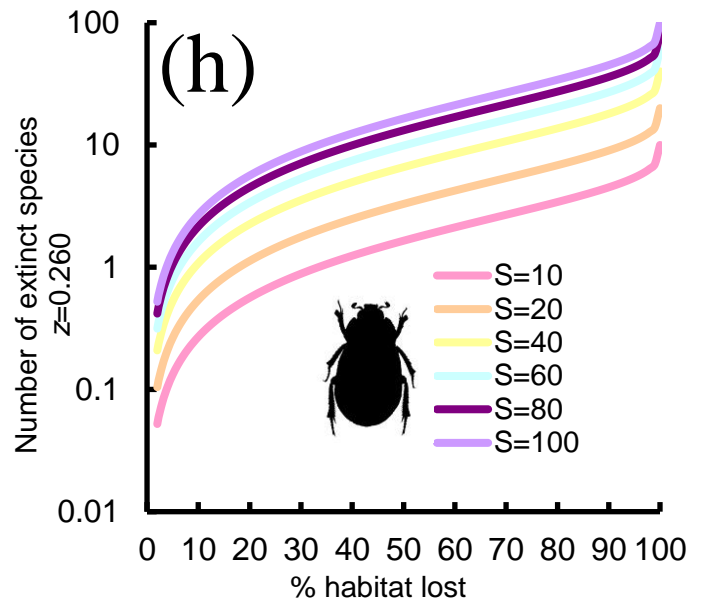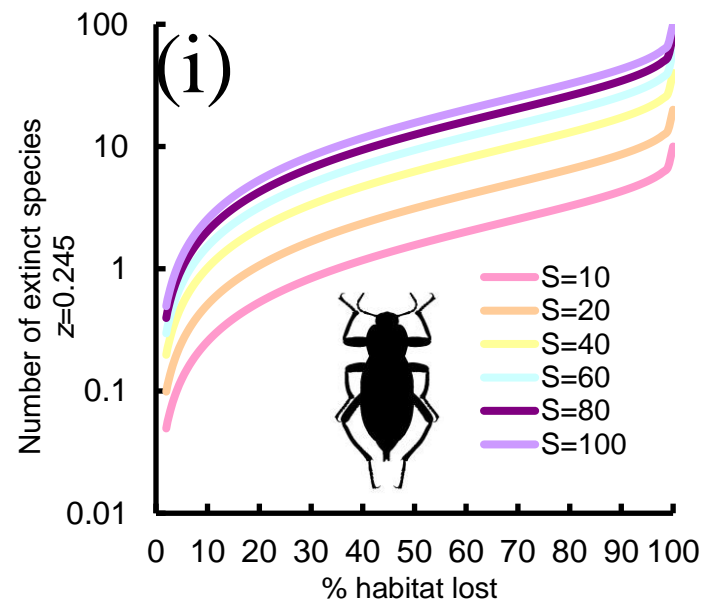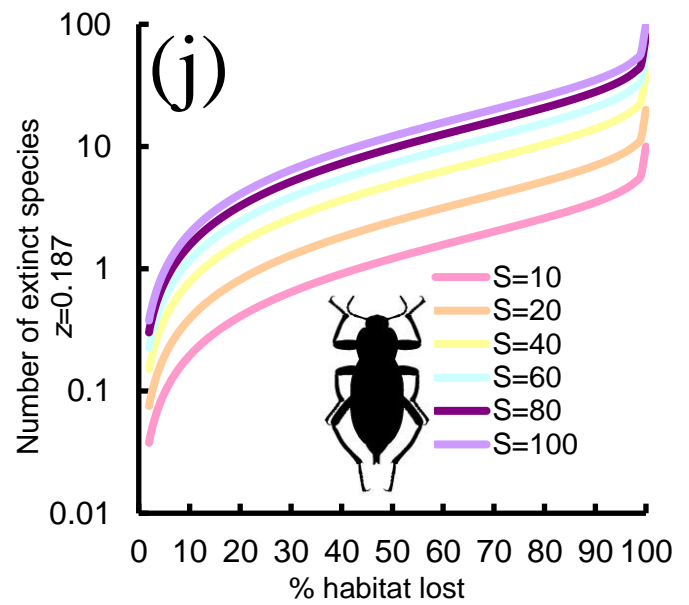

Supplement: Supplementary file 1 [file insects-11-00646-s001.pdf]
